# Motivation Profiles Towards Academic Reading, Substance-Related and Problematic Behaviors in Future Teachers

**DOI:** 10.3390/ejihpe16090124

**Published:** 2026-08-25

**Authors:** María de la Rocha Díaz, Inmaculada Méndez, Nuria Antón-Ros, Cecilia Ruiz-Esteban

**Affiliations:** 1 Department of Didactics of Language and Literature, Faculty of Education, Campus Regional Excellence Mare Nostrum, University of Murcia, 30100 Murcia, Spain; mariarocha@um.es; 2 Department of Evolutionary and Educational Psychology, Faculty of Education, Campus Regional Excellence Mare Nostrum, University of Murcia, 30100 Murcia, Spain; 3 Department of Developmental Psychology and Didactics, Faculty of Education, University of Alicante, 03690 Alicante, Spain; nuria.anton@ua.es; 4 Department of Evolutionary and Educational Psychology, Faculty of Psychology and Speech Therapy, Campus Regional Excellence Mare Nostrum, University of Murcia, 30100 Murcia, Spain

**Keywords:** university, motivation towards reading, substance, problematic behavior, teacher training

## Abstract

Background: Motivation for academic reading is a relevant component of initial teacher training. However, little is known about whether distinct motivational profiles exist in association with patterns of substance use and problematic behaviors among prospective teachers. Methods: A cross-sectional study was conducted with 289 students (77.5% female; aged 18–34) enrolled in Early Childhood Education and Primary Education degree programs. Motivation for academic reading was assessed using the Academic Reading Motivation Scale (EMLA), while substance- and behavior-related problems were evaluated using the MULTICAGE CAD-4 questionnaire. Latent profile analysis was used to identify academic reading motivation profiles, and multivariate and post hoc analyses were performed to examine differences in scores across profiles. Results: Three motivational profiles were identified, characterized by high, intermediate, and low levels of academic reading motivation. Significant differences between profiles were observed regarding problems related to alcohol, drug use, gambling, video games, and compulsive spending. Conclusions: Academic reading motivation profiles were associated with various screening indicators for substance use and problematic behaviors. Given the cross-sectional and self-report nature of the study, these findings should not be interpreted in terms of causality. The results highlight the potential value of jointly considering academic motivation and health-related behavioral indicators in future teacher training.

## 1. Introduction

### 1.1. Academic Reading Motivation in Teachers in Training

Various approaches to influencing motivation can be found in the literature. For our study, we relied on the expectancy-value model proposed by Eccles and Wigfield (2002). This model integrates contributions from classical motivational perspectives—such as self-efficacy (Bandura, 1997)—and aspects of Deci et al. (1999) self-determination theory (SDT). Thus, expectancy-value theory falls within the tradition of achievement motivation, as it posits that the choice of, persistence in, and engagement with an activity depend fundamentally on two major sets of beliefs: expectations of success and the subjective value assigned to the task (Eccles & Wigfield, 2023). Task value comprises the following components: importance, interest, utility, and cost. The concept of interest draws upon the work of Deci et al. (1999): it is the intrinsic valuing of a task by an individual, which involves psychological factors that positively influence performance.

The initial training for early childhood and primary school teachers builds the fundamental foundation for strong education systems. A critical component of this training is promoting motivation for academic reading, which is indispensable for both the acquisition of specialized pedagogical and didactic knowledge and the development of the critical thinking skills essential for a successful teaching career (Guthrie et al., 2004; Lundahl et al., 2017). Recent evidence indicates that university students’ engagement with academic reading is shaped by their attitudes towards assigned texts, the perceived reading difficulty, and their motivational orientation (Eriksson, 2023; Méndez et al., 2025).

Person-centered studies in higher education have likewise identified differentiated motivational and self-regulatory profiles associated with learning strategies, academic engagement, and well-being (Méndez et al., 2025; Zhao & Ling, 2022). Therefore, the identification of these profiles (Ratelle et al., 2007; de la Rocha Díaz et al., 2020; Zhao & Ling, 2022) would allow for the design of specific pedagogical strategies aimed at improving reading, which is so essential to teaching practice (Guthrie et al., 2004). In addition, understanding these motivational profiles can be very useful for subsequent professional development, especially to prevent superficial learning and the avoidance of complex tasks (Ruiz-Esteban et al., 2018).

### 1.2. Substance Use and Other Associated Problematic Behaviors

Substance use and other problematic behaviors—such as excessive mobile phone use, gaming, and compulsive shopping—are becoming increasingly common among young people and are linked to academic performance and, consequently, to psychological well-being (Müller et al., 2015; de la Rocha Díaz, 2022), although the magnitude and direction of these relationships vary across behaviors and study designs (Bulfone et al., 2025; Paterna et al., 2024). Various studies have shown that certain problematic behaviors center around everyday activities such as gambling, compulsive spending, or sexual activity. These activities can stimulate the reward system in the brain in a similar way to substance use. that is, causing dependence and a loss of control (Echeburúa, 1999; Echeburúa & De Corral, 1994; Young, 1999). Therefore, these behaviors may share the same neurological mechanisms as substance addictions (Ruiz Sánchez de León & Pedrero Pérez, 2019). Although the distinction is complex, it is necessary to distinguish normal behaviors from problematic ones (Cascano & Veiga, 2018), especially to prevent them from becoming chronic and having serious personal, social, and professional consequences, among other impacts (Carbonell, 2020).

Therefore, problematic behaviors refer to those that interfere with the performance of daily activities. When these patterns become excessive, they can cause damage similar to that caused by substance use as they are also persistent behaviors that are difficult for the individual to resist and can cause significant personal, social, family, and professional impacts, leading to decreased functioning and quality of life (American Psychiatric Association, 2022; Black, 2022; Estupiñá et al., 2024; Moreira et al., 2023).

#### 1.2.1. Problematic Smartphone and Internet Use

Problematic smartphone use is the excessive and uncontrollable use of mobile devices, which affects daily life, relationships, and academic and work responsibilities. A systematic review and meta-analysis found a small negative association between problematic smartphone use and academic achievement (Paterna et al., 2024). Several studies have found that excessive use of social media or mobile games activates reward circuits in the brain that are linked to dopamine, similar to the use of substances (Tymofiyeva et al., 2020). A meta-analysis found that about 23% of young people worldwide reported problematic smartphone use (Sohn et al., 2019). The greater vulnerability of the adolescent population appears to be mainly due to brain development, emotional sensitivity, and identity formation (Billieux, 2012). With regard to gender, it has been shown that a higher proportion of women show this behavior, which may be due to the fact that they mainly use these devices for the purpose of socializing compared to men who tend to use them mainly for video games or to search for information (Elhai et al., 2017; Lopez-Fernandez, 2017).

#### 1.2.2. Problematic Gaming

Pathological gambling consists of persistent, abusive, and maladaptive behaviors. An individual with problematic gambling behavior exhibits a reduced capacity to inhibit gambling-related motivational impulses due to a domain-specific pattern of temporal discounting, leading them to obtain immediate, small-magnitude rewards at the expense of larger, delayed rewards (Weinsztok et al., 2021). It can have serious family and professional consequences, and can lead to mental health problems, legal problems, etc. (Petry et al., 2014). The diagnostic criteria are clearly defined in the DSM (American Psychiatric Association, 2022), which, unlike problematic smartphone use, has led to the development of various treatment options. People between the ages of 18 and 35 are the most commonly affected, although it can also affect older people (Calado & Griffiths, 2016), and is more common in men than women (Kessler et al., 2008), which may be due to men’s tendency to have greater impulsivity or an interest in sports betting or poker (Chóliz, 2016). However, age and gender should not be presented as isolated or deterministic explanatory factors (Moreira et al., 2023).

#### 1.2.3. Compulsive Spending

On the other hand, compulsive buying is an action that begins as a daily activity, shopping, which becomes problematic when it is repeated continuously and uncontrollably. People often experience a loss of control and a persistent preoccupation with buying. Furthermore, obtaining the immediate gratification that drives compulsive buying can influence the perceived cost compared to that of a sustained academic activity, such as reading (Muenks et al., 2023). This problem ends up affecting multiple areas of life, such as relationships, finances, mental health, etc. (Echeburúa, 1999; Martín-Ríos & Hu-Hai, 2023; Moral Jiménez & Olivares San Emeterio, 2024). It has been shown that this disorder can affect anyone, regardless of their financial situation, so economic resources are a factor but do not determine whether someone will develop the disorder (Black, 2022; Müller et al., 2023). It is very common among young people, which has been greatly exacerbated by the continuous availability of products and stimulation from online platforms (Sol-Gámez et al., 2025). It usually begins between the ages of 18 and 30 (Behar, 2018; Díez Marcet et al., 2016), and is especially common in the university population (Ruiz-Olivares et al., 2010). In terms of gender, women are more commonly affected; they perceive it as a way of coping with stress or anxiety as it usually provides temporary emotional relief (Mueller et al., 2011), and can sometimes increase self-esteem and self-concept, and therefore mood (Behar, 2018). However, the research on potential interventions remains limited and heterogeneous (Müller et al., 2023).

### 1.3. The Present Study

Temporal discounting is the preference for immediate rewards over larger delayed rewards. This preference is associated with both substance-related issues and other behavioral problems, although the strength of this association varies for different behaviors (Exum et al., 2023): it is strongest in the cases of gambling and video gaming disorders, and less pronounced regarding problematic internet or smartphone use (Weinsztok et al., 2021).

According to the expectancy-value model (Eccles & Wigfield, 2023), engagement in highly reinforcing alternative activities rather than academic reading may be associated with the differences in the assigned value and perceived cost. Reading requires an investment of time and sustained cognitive effort, as well as the postponement of other activities (Schwinger et al., 2012). Consequently, engagement in alternative behaviors that are competing for these resources may increase the perceived opportunity costs of academic reading. The valuation of rewards, concurrent demands, and self-regulation difficulties may link substance-related and other problematic behaviors to an individual’s distinct expectation, interest, importance, utility, and cost configuration (Muenks et al., 2023).

In the context of teacher training, various studies have shown that future teachers may have different motivational profiles for academic reading (de la Rocha Díaz et al., 2020; Santos Díaz et al., 2021). Thus, differences in motivation for academic reading can be understood not only in terms of whether students enjoy reading, but also whether they expect to succeed, whether they consider academic reading as important and useful, and, above all, whether they perceive it as requiring considerable effort compared to other activities (de la Rocha Díaz, 2022; Schwinger et al., 2012). However, it is unknown how these motivational profiles are distributed among future teachers and whether they are related to substance use or other problematic behaviors.

### 1.4. Aim and Contribution of the Study

This study aimed to identify patterns of association between academic reading motivation and substance-related and other problematic behaviors. The following research questions were addressed:

- RQ1. What distinct academic reading motivation profiles are present among future teachers based on expectations, interest, importance, utility, and cost?
- RQ2. Does the risk of substance-related and other problematic behaviors differ across the identified academic reading motivation profiles?

The analyses identified three distinct academic reading motivation profiles characterized by high, intermediate, and low levels of motivation. The low-motivation profile showed higher screening scores for alcohol and drug-related problems, problem gambling, video game-related issues, and compulsive shopping; conversely, no significant differences were found between the profiles regarding problematic smartphone or Internet use. These findings should be interpreted as associations rather than causal effects.

## 2. Materials and Methods

### 2.1. Participants

Participants were recruited using non-probability convenience sampling from Primary Education and Early Childhood Education bachelor programs. Eligibility was based on enrolment in either of these programs during the data collection period. A total of 431 students met the eligibility criteria and were invited to participate; 289 provided informed consent and fully completed the instruments, yielding a response rate of 67.1%.

The participants ranged in age from 18 to 34 years (mean age = 21.59 years, SD = 3.52). The majority of the participants were women (77.5%), while men accounted for 22.5%.

### 2.2. Instruments

The following measurement instruments were used in the study. First, a sociodemographic scale was administered to obtain information about age, sex, and place of birth. Second, the Academic Reading Motivation Scale (EMLA) by Muñoz Valenzuela et al. (2012) was administered to gather information about motivation toward academic reading. This scale consists of 27 items that are scored from 1 (total disagreement) to 6 (total agreement) and is based on the expectancy-value model of Eccles and Wigfield (2002). The items cover the following dimensions: Expectations, which is the reader’s perception of whether they will be capable of successfully completing the task (for example, “When reading academic texts, I manage to grasp the central ideas”); Interest, which focuses on the affective component of the task, that is, the enjoyment from performing the task (for example, “I enjoy reading academic texts related to my degree”); Importance, which refers to the relevance of performing the task (for example, “For me, it is important to have completed the required readings before class (when applicable)”); Utility, which refers to the perceived usefulness of academic reading for present or future academic and professional goals (for example, “I consider understanding academic texts highly useful”); and Cost, which is the perceived limitations that performing the task will have on performing other activities (for example, “I am capable of setting aside other interests and committing myself until I properly finish reading a text”). In this study, the Cronbach’s alpha reliability values for these subscales were acceptable, ranging from 0.76 to 0.91, and the value for the total scale was 0.97.

Thirdly, Rodríguez-Monje et al.’s (2019) version of the MULTICAGE CAD-4 questionnaire was administered. It is a screening tool used to assess the risk associated with substance use and other problematic behaviors. This instrument comprises 24 dichotomous items that analyze the following subscales: Alcohol Use, which includes abusive intake of alcoholic beverages (e.g., “Have you ever thought that you should drink less?”); Drug Use, which measures aspects of problematic drug use (e.g., “Have you ever thought that you should use fewer drugs?”); Gambling, which focuses on gambling-related behaviors (e.g., “Have you experienced psychological, familial, economic, or work-related problems due to gambling?”); Smartphone Use, which analyzes problematic mobile phone use (e.g., “If you don’t have your mobile phone one day, do you feel uncomfortable or as if something is missing?”); Internet Use, which measures excessive internet use (e.g.,: “Do you find it hard to stay away from the internet for several consecutive days?”); and Video Gaming, which measures problematic behaviors related to video console or computer games (e.g., “Does your family complain that you spend too much time playing video consoles or computer games?”). In this study, the Kuder–Richardson (KR-20) coefficient values obtained for the subscales ranged from 0.74 to 0.78, and the value for the total scale was 0.83.

The Compulsive Spending subscale from the MULTICAGE CAD-4 version by Pedrero-Pérez et al. (2007) was also included to evaluate uncontrolled compulsive spending and financial issues due to compulsive purchases (e.g., “Have you had problems with your family due to your excessive spending and lack of control over money?”). The KR-20 for this subscale in our study was 0.72.

### 2.3. Procedure

Prior to initiating the study, formal approval was obtained from the Research Ethics Committee of the University of Murcia in April 2020 (ID: 2819/2020). Following this authorization, the faculty responsible for courses within the Department of Language and Literature Didactics—which is part of the Primary Education and Early Childhood Education degree programs at a university in southwestern Spain—were contacted to facilitate the recruitment of students and the administration of the assessment instruments. Participants were recruited from students enrolled in these degree programs during the 2022–2023 academic year. During an in-person session lasting approximately 50 min during a class, students were informed of the research objectives and purpose, as well as the anonymous and confidential nature of the study. Informed consent was obtained from the participants, who took part voluntarily without receiving any reward and had the right to withdraw from the study at any time. Once collected, the data were entered into a secure database; to ensure that individual responses could not be linked to a specific person, each participant was assigned an alphanumeric code.

### 2.4. Data Analysis

First, we calculated the Spearman correlation coefficients between the dimensions of motivation for academic reading and the dimensions of risk associated with substance use and other problematic behaviors. The Spearman coefficient was selected after examining the distribution of the variables as we observed deviations from normality, characterized by high skewness and kurtosis values. The effect sizes were interpreted according to Cohen (1988): 0.10–0.29 (small), 0.30–0.49 (moderate), and ≥0.50 (large). Next, the academic reading motivation dimensions were standardized, and a Latent Profile Analysis (LPA) was performed using robust maximum likelihood estimation (MLR); in the analyses, we specified 500 random starts for the initial stage and 100 final-stage optimizations, with 20 initial-stage iterations, and we inspected TECH8 to verify convergence and replication of the best log-likelihood solution. The default latent profile model assumes class-invariant variances and within-profile covariances equal to zero. To evaluate the sensitivity of the solution to this specification, we also examined models that allow the indicator variances to vary across profiles while keeping the within-profile covariances at zero. To identify participant groups, the optimal model was selected based on the joint consideration of the following criteria based on fit indices, as well as their stability and interpretability: low Akaike Information Criterion (AIC) and Bayesian Information Criterion (BIC), Vuong–Lo–Mendell–Rubin Likelihood-Ratio Test (LRT) *p* < 0.05, and Bootstrap Likelihood-Ratio Test (BLRT) *p* < 0.05. The entropy values and theoretical interpretability of the profiles were also considered, and each profile was required to include at least 25 subjects from the sample (Tein et al., 2013) because profiles with too few subjects would result in low statistical power (Type II error) and low generalizability. Subsequently, a Multivariate Analysis of Variance (MANOVA) was used to examine differences in the substance use and other problematic behavior subscale scores across the identified profiles. Since there was no multivariate normality, we used Pillai’s trace as the primary multivariate statistic due to its relative robustness to deviations from multivariate normality and covariance homogeneity. In addition, given that Levene’s tests indicated heterogeneity of variances in the dependent variables, Games–Howell post hoc comparisons were used. Partial eta-squared (η*p*^2^) values were used to quantify the overall effect size for each addiction dimension. When statistically significant differences emerged via post hoc tests, Cohen’s d coefficients were calculated to specify the magnitude of the difference, which were classified as follows: 0.20–0.49 (small effect), 0.50–0.79 (moderate effect), and ≥0.80 (large effect). All analyses were performed using the statistical software packages SPSS (v.28.0) and Mplus (v.8.4).

## 3. Results

The participants were enrolled in either the Primary Education (53.3%, *n* = 154) or Early Childhood Education bachelor’s degree program (46.7%, *n* = 135). The majority (95.5%, *n* = 276) were Spanish, while 4.5% were born outside of Spain.

The analyses identified three distinct academic reading motivation profiles: high, intermediate, and low levels of motivation. The profiles differed significantly in several screening indicators related to substance use and other problematic behaviors. In general, participants with the low-motivation profile showed higher scores for problems related to alcohol, drug use, gambling, video games, and compulsive spending, whereas no significant differences were observed between profiles regarding smartphone or Internet use. The detailed results of the assessment, latent profile, and between-group comparison analyses are presented below.

The Spearman correlations (Table 1) indicated significant negative associations between all academic reading motivation dimensions and alcohol-related problems, drug use, gambling, and compulsive spending. In contrast, problematic smartphone use, internet use, and video gaming were not significantly correlated with the five academic reading motivation dimensions.

These consistent inverse relationships across all motivational dimensions prompted a latent profile analysis.

The model fit indices for the latent profile solutions are presented in Table 2. The five- to seven-profile solutions were rejected because, although they yielded lower AIC and BIC values, they included at least one group with fewer than 25 cases; such a small group size would result in low statistical power (Type II error) and limited generalizability (Tein et al., 2013). The four-profile model was also rejected because it included a latent profile with a non-significant LRT. Among the remaining models, the three-profile model provided the best fit, as it showed lower AIC and BIC values alongside higher entropy, indicating a clear delineation of substantially meaningful and interpretable profiles.

**Figure 1:**
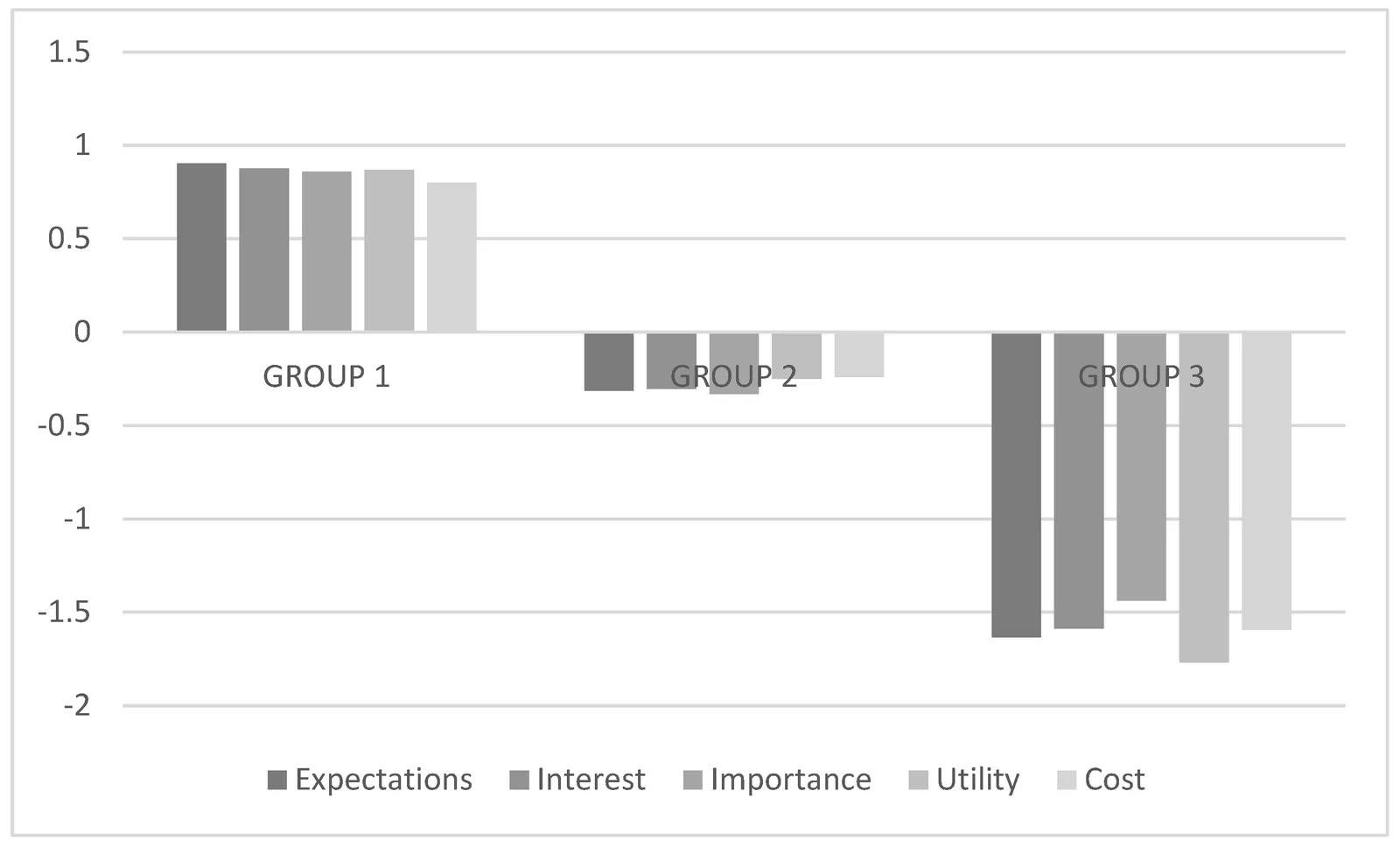
Graphical representation of the three-group model.

Figure 1 depicts the three emergent academic reading motivation profiles: (a) a group of 115 students (39.8%), characterized by consistently high values across all academic reading motivation dimensions (Group 1); (b) a group of 140 students (48.4%) that exhibited intermediate values in the different reading motivation dimensions (Group 2); and (c) a group of 34 students 3 (11.8%) that showed uniformly low values across all reading motivation dimensions (Group 3).

Regarding differences in problematic behavior scores across the different academic reading motivation profiles, the MANOVA results revealed significant differences across most problematic behavior subscales (Pillai’s trace = 0.17, (*F*(_14,286_) = 3.60, *p* < 0.001, *np*^2^ = 0.08; Table 3). Table 4 reports the Cohen’s *d* values for the post hoc comparisons (Games–Howell) of significant dimensions.

Post hoc analyses for alcohol abuse or dependence indicated significant differences between the groups (*F*(_2,286_) = 10.71, *p* < 0.001, η^2^ = 0.07). Students in Group 3 (M = 1.16, SD = 1.05) scored significantly higher than those in Group 1 (M = 0.41, SD = 0.81; *p* < 0.001, *d* = −0.87) and Group 2 (M = 0.77, SD = 0.91; *p* = 0.004, *d* = −0.42). Additionally, Group 2 scored higher than Group 1 (*p* = 0.005, *d* = −0.42).

For other drugs, there were significant differences between the groups (*F*(_2,286_) = 6.70, *p* = 0.001, η^2^ = 0.05). Group 3 (M = 0.58, SD = 1.10) scored significantly higher than Group 1 (M = 0.12, SD = 0.42; *p* < 0.001, *d* = −0.72) and Group 2 (M = 0.21, SD = 0.66; *p* = 0.009, *d* = −0.48). No significant differences were observed between Group 1 and Group 2.

Regarding problematic gambling, significant differences between the groups were found (*F*(_2,286_) = 11.16, *p* < 0.001, η^2^ = 0.07). Group 3 (M = 0.64, SD = 1.05) scored significantly higher than Group 1 (M = 0.11, SD = 0.52; *p* < 0.001, *d* = −0.78) and Group 2 (M = 0.32, SD = 0.48; *p* = 0.016, *d* = −0.51). Group 2 also scored higher than Group 1 (*p* = 0.017, *d* = −0.42).

There were significant differences in scores on the Video gaming subscale between the groups (*F*(_2,286_) = 4.80, *p* = 0.009, η^2^ = 0.03). Group 3 (M = 0.71, SD = 1.15) scored significantly higher than Group 1 (M = 0.25, SD = 0.77; *p* = 0.010, *d* = −0.53) and Group 2 (M = 0.27, SD = 0.72; *p* = 0.012, *d* = −0.54). No significant differences were found between Group 1 and Group 2.

Regarding compulsive spending, there were significant differences between the groups (*F*(_2,286_) = 8.17, *p* < 0.001, η^2^ = 0.05). Group 3 (M = 0.84, SD = 1.06) scored significantly higher than Group 1 (M = 0.27, SD = 0.77; *p* = 0.001, *d* = −0.68) and Group 2 (M = 0.28, SD = 0.66; *p* < 0.001, *d* = −0.74). No significant differences were observed between Group 1 and Group 2.

Finally, regarding smartphone use and internet use, no significant differences were detected between the groups (*F*(_2,286_) = 0.25, *p* = 0.776 and *F*(_2,286_) = 0.18, *p* = 0.833, respectively).

## 4. Discussion

The study identified three distinct profiles for academic reading motivation: a group with high values in all dimensions of motivation for academic reading (Group 1), a group with intermediate values (Group 2), and a group with low values (Group 3). These groups showed differences in scores for most of the substance use and problematic behavior subscales.

It is worth noting that the students with low motivation for academic reading (Group 3) exhibited higher levels of risk regarding problematic substance use (alcohol and drugs) and other problematic behaviors, such as problematic gambling, video gaming, and compulsive spending. According to the expectancy-value model (Eccles & Wigfield, 2023), engagement in alternative activities—such as substance use and other highly reinforcing problematic behaviors—may influence the value and perceived cost assigned to academic reading (Muenks et al., 2023). According to this model, our results indicate that low-motivation-profile students may view academic reading as requiring time and sustained cognitive effort, in contrast to the immediate gratification offered by certain alternative behaviors. This aligns with the research on temporal discounting, which points to a greater preference for immediate rewards—a pattern often observed in substance use and other problematic behaviors (Exum et al., 2023).

Furthermore, from the perspective of SDT, the interest component of academic reading motivation is closely related to intrinsic motivation, whereas expectations of success are conceptually linked to perceived competence; meanwhile, the importance and utility attributed to reading may reflect more internalized reasons for academic engagement (Ryan & Deci, 2020). Consequently, the coexistence of a low academic reading motivation profile with higher scores for various problematic behaviors is consistent with lower self-determined engagement and weaker self-regulation ability.

Similarly, Group 2 also showed higher levels of alcohol abuse and pathological gambling than Group 1, suggesting that these behaviors may co-occur with less favorable academic reading motivation profiles (Bulfone et al., 2025; Moreira et al., 2023). This pattern is consistent with the findings of Muenks et al. (2023), who found that problematic behaviors occur when there are competing demands on time, attention, and self-regulatory resources, potentially leading to a higher perceived cost for sustained academic activities such as reading. This interpretation is consistent with the evidence regarding delay discounting in problematic activities and substance-related behaviors (Exum et al., 2023; Weinsztok et al., 2021); however, the cross-sectional design of these studies prevented the determination of the direction of the association.

On the other hand, it should be noted that no significant differences were observed between the groups in terms of problematic smartphone and internet use, which is in line with previous literature (Jiang et al., 2024; Liu et al., 2025). This may reflect domain specificity; unlike substance use or problematic behaviors such as pathological gambling, excessive digital media use can coexist with academic routines and may therefore show weaker associations with academic motivation (Paterna et al., 2024; Weinsztok et al., 2021). Our findings support a potential association between certain problematic behaviors and motivation for academic reading.

### Strengths, Limitations, and Future Research

The study’s strengths include the following. First, the robust, person-centered latent profile approach made it possible to jointly examine multidimensional patterns of academic reading motivation, rather than considering each dimension in isolation. Second, the use of validated instruments allowed for the assessment of academic reading motivation and the risk of various problematic behaviors and substance use-related issues. Third, it enabled a detailed analysis of differences between profiles regarding the dimensions of academic reading motivation.

The results of this study have several practical implications. Within university settings, initiatives aimed at the early detection of substance use and other problematic behaviors should be promoted, and academic motivation should be fostered alongside well-being indicators. Consequently, students demonstrating lower motivation for academic reading could represent a target group for broader initiatives focused on prevention, education, and well-being.

Several limitations must also be considered. The cross-sectional design and reliance on self-reported measures preclude us from drawing conclusions regarding the temporal sequence or causality. The use of a non-probability convenience sample focused on a single university may limit the generalizability of the findings to other student populations. Furthermore, the MULTICAGE CAD-4 questionnaire is a screening instrument; its results should not be interpreted as clinical diagnoses but rather as indicators of risk for substance use or other problematic behaviors. Finally, potentially relevant variables—such as mental health, personality, stress levels, impulsivity, academic performance, and direct indicators of Self-Determination Theory (SDT) constructs—were not assessed and could have influenced the observed associations.

Future research should attempt to replicate these profiles using larger, more diverse samples across multiple institutions. Longitudinal designs are needed to clarify the temporal sequence, while qualitative or mixed-methods approaches could help identify the mechanisms that precipitate or protect against substance use, problematic behaviors, and declines in motivation. Additionally, studies incorporating direct measures of autonomous and controlled motivation, basic psychological needs, academic performance, and behavioral indicators or complementary clinical measures would allow for a more detailed assessment of the motivational processes proposed by SDT.

## 5. Conclusions

The study highlighted several aspects that should be considered in the training of future teachers. It is essential to prioritize the early detection of substance use and other problematic behaviors, which may coexist with less favorable academic reading motivation profiles. Similarly, interventions should focus on prioritizing self-regulation techniques together with educational strategies that promote autonomy and intrinsic interest in academic reading. In addition, universities should promote programs aimed at increasing interest in academic reading by enhancing their reading commitment.

## Figures and Tables

**Table 1:** Spearman Correlation between the variables of study.

Variable	Expectations	Interest	Importance	Utility	Cost
Alcohol Use	−0.558 **	−0.482 **	−0.497 **	−0.528 **	−0.493 **
Drug Use	−0.126 *	−0.165 **	−0.157 **	−0.105	−0.125 *
Gambling	−0.293 **	−0.254 **	−0.333 **	−0.286 **	−0.275 **
Smartphone Use	0.044	0.004	0.001	0.086	0.003
Internet Use	0.000	−0.008	−0.052	0.048	0.007
Video Gaming	−0.330 **	−0.255 **	−0.286 **	−0.317 **	−0.315 **
Compulsive Spending	−0.343 **	−0.296 **	−0.272 **	−0.320 **	−0.304 **

Note. * *p* < 0.05, ** *p* < 0.005.

**Table 2:** Fitting latent profile models.

Models	AIC	BIC	BIC-Adjusted	LRT	LRT-Adjusted	BLRT	Entropy	Size Group
1	4115.72	4152.39	4120.68	-	-	-	-	289
2	3437.81	3496.47	3445.73	0.036	0.0392	<0.001	0.857	123
**3**	**3064.26**	**3144.92**	**3075.16**	**0.016**	**0.0182**	**<0.001**	**0.915**	**34**
4	2943.75	3046.41	2957.61	0.122	0.1268	<0.001	0.889	19
5	2873.14	2997.80	2889.98	0.034	0.0374	<0.001	0.880	18
6	2811.76	2958.42	2831.58	0.047	0.0506	<0.001	0.890	3
7	2774.81	2943.46	2797.59	0.026	0.0290	<0.001	0.907	3

Note. Akaike Information Criterion (AIC); Bayesian Information Criteria (BIC); LRT = Vuong–Lo– Mendell–Rubin Likelihood-Ratio Test; BLRT = Bootstrap Likelihood Ratio Test. Size group: Number of participants in the smallest profile. The values in bold show the selected model.

**Table 3:** Means and standard deviations in the three academic reading motivation groups regarding each of the substances and problematic behaviors.

	Group 1		Group 2		Group 3		Significance Statistics		
Dimensions	M	SD	M	SD	M	SD	*F*_(2,286)_	*p*	η^2^
Alcohol Use	0.41	0.81	0.77	0.91	1.16	1.05	10.71	<0.001	0.07
Drug Use	0.12	0.42	0.21	0.66	0.58	1.10	6.70	0.001	0.05
Gambling	0.11	0.52	0.32	0.48	0.64	1.05	11.16	<0.001	0.07
Smartphone Use	2.07	1.33	1.96	1.29	2.05	1.30	0.25	0.776	<0.01
Internet Use	1.55	1.23	1.61	1.31	1.70	1.33	0.18	0.833	<0.01
Video Gaming	0.25	0.77	0.27	0.72	0.71	1.15	4.80	0.009	0.03
Compulsive Spending	0.27	0.77	0.28	0.66	0.84	1.06	8.17	<0.001	0.05

Note. Group 1: Students with high values in motivation for academic reading; Group 2: Students with intermediate values in motivation for academic reading; Group 3: Students with low values in motivation for reading. Partial eta squared values (η*p*^2^).

**Table 4:** Cohen’s *d* indices for post hoc contrast groups.

Dimensions		1-2	1-3	2-3
Alcohol Use	*d*	−0.42	−0.87	−0.42
Drug Use	*d*	−0.15	−0.72	−0.48
Gambling	*d*	−0.42	−0.78	−0.51
Smartphone Use	*d*	0.08	0.02	−0.08
Internet Use	*d*	−0.05	−0.11	−0.06
Video Gaming	*d*	−0.02	−0.53	−0.54
Compulsive Spending	*d*	−0.01	−0.68	−0.74

## Data Availability

Data are unavailable due to privacy or ethical restrictions. The datasets generated and analyzed during the current study are available from the corresponding author on reasonable request.

## Abbreviations

The following abbreviations are used in this manuscript:

AIC	Akaike Information Criterion
BIC	Bayesian Information Criterion
LRT	Latent Profile Ratio
BLRT	Bootstrap Likelihood Ratio Test.
SDT	Self-Determination Theory
MANOVA	Multivariate Analysis of Variance
